# The Effect of Mobile Health (m-Health) Education Based on WANTER Application on Knowledge, Attitude, and Practice (KAP) Regarding Anemia among Female Students in a Rural Area of Indonesia

**DOI:** 10.3390/healthcare10101933

**Published:** 2022-10-02

**Authors:** Puspa Sari, Dewi Marhaeni Diah Herawati, Meita Dhamayanti, Tisa Layalia Hanifah Ma’ruf, Dany Hilmanto

**Affiliations:** 1 Doctoral Study Program, Faculty of Medicine, Universitas Padjadjaran, Bandung 45363, West Java, Indonesia; 2 Department of Public Health, Faculty of Medicine, Universitas Padjadjaran, Bandung 45363, West Java, Indonesia; 3 Department of Child Health, Hasan Sadikin Hospital, Faculty of Medicine, Universitas Padjadjaran, Bandung 45363, West Java, Indonesia; 4 Master of Public Health Study Program, Faculty of Medicine, Universitas Padjadjaran, Bandung 45363, West Java, Indonesia

**Keywords:** anemia, adolescent girls, mobile health education, booklet, knowledge, attitude, practice (KAP)

## Abstract

Female students, as adolescent girls, are more at risk of anemia because of high nutritional requirements. Health education through mobile applications influences the knowledge, attitude, and practice (KAP) of anemia in adolescent girls. Based on our previous study, several adolescents experienced anemia. This study aims to analyze the effect of health education through mobile applications, namely the WANTER application, on increasing KAP. This study was quasi-experimental with a pretest–posttest design; the sample was divided into an intervention (WANTER application) and a control group (booklet). The study was conducted in a rural area of Indonesia. There were 162 adolescent girls included in the intervention group and as many as 115 adolescent girls in the control group. Our study showed that adolescents’ knowledge and attitude increased significantly in three months after the intervention of WANTER and booklets toward preventing anemia with *p* < 0.001; however, there was no significant difference in KAP between the control and intervention groups. In addition, there was no improvement in practice, either in the control or intervention groups. Knowledge, attitudes, and practices to prevent anemia need to be continuously improved. Health education through appropriate media for adolescents is very important to make interventions more effective.

## 1. Introduction

Anemia is a condition where the oxygen-carrying capacity in the blood is insufficient [1]. The normal level of hemoglobin in adolescent girls is 12 g/dL [2]. Several factors can cause anemia, such as deficiency of vitamin A, vitamin B12, folate, and iron, chronic inflammation, parasitic infections, and congenital conditions. However, due to a lack of iron required for the production of red blood cells, iron deficiency anemia (IDA) is a prominent cause throughout the world. Anemia can lead to increased pregnancy complications, such as low birth weight (LBW), premature birth, and neonatal death [3]. Anemia also has a negative impact on physical abilities, development, performance, and immunity, and causes potentially long-term effects in women of childbearing age [4]. Anemia affects 33% of women of reproductive age in 2016, with Asia and Africa having the greatest prevalence. According to the 2018 Indonesian National Health Survey, the prevalence of anemia in people aged 5 to 14 and 15 to 24 was 26.8% and 32%, respectively [5]. Our previous study revealed that the prevalence of anemia among female students in Soreang District as a rural area was 14.3% [6]. The government runs a program of giving iron supplementation to overcome IDA in adolescents. Iron supplements are given to female students at junior high school and high school, once a week. This initiative is, however, viewed as being less successful due to the continued low consumption of iron tablets among female students. Awareness of anemia is one of the influencing variables [7].

Female students are in the transitional stage between childhood and adulthood known as adolescence. Changes in an individual’s physical, biological, hormonal, psychological, behavioral, and sexual maturity are its defining characteristics [8,9]. According to the World Health Organization (WHO), adolescence begins between the ages of 10 of 19 years [10]. Adolescent girls go through a variety of psychological changes in addition to physiological ones, which together can have an impact on body image and how they react to societal pressures. According to a recent study, adolescent girls who go through early menarche may be more inclined to communicate via social media than face-to-face socialization as the latter allows them to offer a more idealized version of themselves [11,12].

Health education through WANTER as m-Health applications can improve health. WANTER can improve communication between adolescents and health workers with the support of a telemedicine system. Adolescents can ask questions about health problems and have consultations. Therefore, health applications are an important part of the relationship between health and technology [13]. It is very important to run a health education program for adolescents as an effort to prevent anemia. Health education in schools plays a major role in improving students’ knowledge, attitude, practice (KAP) [14]. KAP assessment is suitable for evaluating the effectiveness of intervention programs. In addition, it can assess the current knowledge, attitudes, and practices of the target group on a particular topic to detect their needs, problems, and possible barriers before developing and implementing intervention studies. This questionnaire can identify what a female student already knows (Knowledge), how they feel (Attitude), and what they do (Practice). KAP was used to evaluate knowledge about the management of IDA in adolescent girls. This study used the latest KAP questionnaire from the Food and Agriculture Organization of the United Nations (FAO) to be used as a KAP evaluation tool among adolescents. Furthermore, IDA-related attitude and practice questions were used to identify the level of correct attitudes and practices toward health [15]. This study used a health education program through the m-health application, namely *Wanoja Anti-Anemia Pinter Tur Cageur* (WANTER), as an intervention to increase the KAP of anemia among adolescent girls. In Indonesian, *Wanoja Anti-Anemia Pinter Tur Cageur* means a woman without anemia who is healthy and smart. This study aimed to analyze the effect of health education through a mobile application, namely the WANTER application, on increasing KAP.

## 2. Materials and Methods

### 2.1. Design

The study was conducted using a quasi-experiment with a pretest–posttest design. The sample was divided into an intervention group and a control group [16,17,18]. Respondents decided whether to join the intervention or control group. The WANTER application was developed by our team in collaboration with an informatics expert. A health education application, named WANTER, is a form of mobile health, which contains videos about iron deficiency anemia, a body mass index (BMI) calculator, balanced nutrition, and a consultation menu regarding anemia, which are responded by us as application makers.

### 2.2. Participants

The subjects of this study were adolescent girls aged 15–18 years or equivalent to high school students in the Soreang District, Bandung Regency. Respondents were selected based on cluster sampling, consisting of public and private schools in the Soreang sub-district, where Soreang is the city center in the Bandung Regency area. Moreover, the current research is a continuation of previous research [6]. A total of 277 subjects agreed to participate in the study and stated that they understood the purpose of the study. Participants were recruited by the method of filling out a willingness to participate in the research submitted by each teacher in each school. Of these, 162 people agreed to participate in the intervention group. The remaining 115 subjects were the control group. Members of the intervention group were given education through the WANTER application, while the control group was given a booklet. The intervention process was carried out for three months, then a posttest questionnaire was given. The inclusion criteria in this study were adolescent girls who were in grades 10 and 11 of high school/equivalent, willing to participate in the study by signing an informed consent. The exclusion criteria were adolescent girls who were not present during the pretest and posttest questionnaire sessions.

### 2.3. Data Collection

The instrument in this study used a KAP questionnaire (Appendix A) regarding Iron Deficiency Anemia sourced from FAO [19]. The questionnaire was used with FAO’s permission via email. The questionnaire used was tested for validity and reliability, adapted to the cultural customs of the Indonesian people. The questionnaire consists of 8 questions from the knowledge section, 6 questions from the attitude section, and 3 questions from the practice section. The pretest questionnaire was filled in using a Google form, which was filled out by each student starting in March 2022.

After completing the questionnaire, students in the intervention group were given access to install the WANTER application on the Play Store. In contrast, students in the control group were given a booklet on anemia in adolescent girls. The intervention group for three months received health education, consultation, and examination results regarding nutritional status through the application. Posttest questionnaire data were collected in June 2022 with the aim of obtaining differences in the results of the intervention group and the control group.

### 2.4. Data Analysis

KAP Data Analysis (FAO): (1) Knowledge refers to the respondent’s understanding of anemia. The questions given are questions with a choice of answers and a list of answers that can be filled in by the respondent briefly. Then, the researcher assesses the respondents’ answers through a pre-preliminary box analysis. If the question has a single correct answer, the options are “know“ or “Does not know”. If the question has several correct answers, the options are “know“ (if the respondent gives one, some, or all possible correct answers), “Does not know” (if the respondent gives no correct answers), and “Number of correct responses” (to indicate the number of correct answers provided). Respondents who answered “Knows” were given several 1, while those who “do not know” were given several 0. The maximum number for knowledge is 8, according to the number of questions given. (2) Attitude. By asking respondents to rate their level of positivity or negativity, attitudes are measured. The attitude element of the questionnaire asked participants to rate their level of confidence in their abilities to prepare iron-rich meals, as well as their opinions on their taste and likelihood of developing IDA. Participants were categorized as having positive attitudes for analysis if they believe that IDA is a serious disease or that they are likely to contract this disease, feel good or confident about preparing an iron-rich meal, believe that preparing this meal is not difficult, or like the flavor of the meal. Participants were categorized as having negative attitudes if they provided opposing replies or “maybe/not sure” responses. (3) Practice. In the practice section, the first question asked if the participant had eaten any foods high in iron the day before, the second inquired about whether they typically eat fruits high in vitamin C, and the third evaluated if they typically drink tea or coffee. The participants responded to two more questions about daily coffee or tea use and timing of intake if the final two questions were answered as affirmative. Participants were categorized as having healthy habits for analysis if they stated that they had eaten iron-rich meals the day before, typically eat fruits high in vitamin C, or do not typically drink tea or coffee. However, participants who provided the opposite responses had unhealthy habits. Assessment of knowledge, attitudes, and practices can use numbers, percentages, or scores. This study used analysis in the form of numbers, with a maximum value of 8 for knowledge, 6 for attitude, and 3 for practice. This maximum number corresponds to the number of question numbers [19].

Data were analyzed using IBM SPSS software Version 27.0 for Windows. A comparison of general characteristics between the experimental and control groups was measured using the Mann–Whitney test and the chi-square test. A comparison between the level of knowledge, attitudes, and practice of the intervention group and the control group was carried out using the Wilcoxon and Mann–Whitney tests.

### 2.5. Ethical Considerations

Prior to conducting this research, approval was obtained from the Research Ethics Commission of the University of Padjadjaran by the Declaration of Helsinki, and this research was approved with the number 018/UN6.KEP/EC/2022. Participants were briefed on the purpose of the study and informed that they were free to withdraw from the study at any time. Written informed consent was obtained from adolescent girls and also the parents as legal guardian. This research was the follow-up from a previous study that analyzed anemia and the factors that influence anemia in adolescent girls [6].

## 3. Results

### 3.1. Participant Characteristics

General characteristics of the intervention and control groups are shown in Table 1. There were 162 adolescent girls included in the intervention group and as many as 115 adolescent girls in the control group. The number of respondents who were included in the control group was less than the number of the intervention group, because there were several samples in the control group who dropped out, who did not fill out the posttest questionnaire.

There was no significant difference between the two groups regarding father’s education (*p* = 0.52), mother’s education (*p* = 0.256), father’s occupation (*p* = 0.779), mother’s occupation (*p* = 0.604), household monthly income (0.239), and purpose of coming to health services (0.785), but there is a difference based on resources about IDA (*p* = 0.003) from both intervention and control groups. This study showed that there was a significant difference based on the living with/in (*p* = 0.000) from the intervention and control groups, but based on the educational stage (*p* = 0.223), history of hemoglobin test (*p* = 0.85), history of taking blood tablets (*p* = 0.57), anemia status (*p* = 0.597), and experience coming to health services (*p* = 0.785), there were no differences between the two groups.

### 3.2. Comparison of Knowledge, Attitudes, and Practices (KAPs) between Control and Intervention Group

Table 2 showed that there was a significant relationship between knowledge and attitudes before and after the intervention, with *p* < 0.001. An increase in knowledge and attitudes, after three months, in both the intervention and control groups was seen in the median value. However, there was no improvement in practice, either in the control or intervention groups. In addition, there was no significant difference between the control group and the intervention group, either before or after treatment.

## 4. Discussion

Table 1 shows that the characteristics between the intervention and control groups are not significantly different. However, there are significant differences in the variables of living with/in and source information. The majority of adolescents in the intervention group stay together with their families. In addition, the group intervention receives more information regarding anemia from WANTER. Living with parents could influence adolescents to look for information regarding anemia from social media, electronic media, and the environment. In contrast, living in a dormitory could limit access to social media and make them only depend on information from the environment, such as friends and teachers. Therefore, teachers are expected to spread knowledge about anemia and its prevention actively. Health education has become an important part of increasing the knowledge about the prevention of anemia [20,21]. The source information about anemia could be obtained from teachers, health workers, and friends [22]. Adolescent health behavior depends on reliable sources [23]. The inability to access credible, reliable, and accurate information could impact negatively on the knowledge [24].

Table 2 shows that there is no significant difference among the control group regarding KAP, against anemia prevention. After 3 months, the intervention and control group show a significant improvement in knowledge and attitude for preventing anemia with a *p*-value <0.01. Health education is an important factor, as the basis for changing knowledge, attitudes and practices in preventing anemia [25]. A person’s opinion or assessment of matters related to healthcare is the definition of attitude [19], while practice is all activities of people in order to maintain health. Previous research has explained that knowledge, attitudes, and practices (KAPs) on anemia can improve health behavior [26]. Other studies have explained that attitude and practice regarding anemia are significantly correlated with hemoglobin levels [27]. Health education effectively improves knowledge, attitudes, and practices within a sufficient time. Although most studies have revealed that the adequate time is six months or more, three months of intervention improved knowledge and attitudes, except practice. Counseling programs for healthy adolescents at school or in the community are important. Health education through WANTER as m-health education and booklets is needed at the moment. In other studies, it is possible to explore education through m-Health [28,29,30]. WANTER will help adolescents monitor health, intake nutrition, and source information on health. In addition to WANTER, another media that can be used to educate adolescents is a booklet, for obtaining knowledge regarding anemia. Booklets can encourage adolescent girls to find out about nutritious food, counting methods of body mass index (BMI), and health. Another study concluded that booklets had a significant effect on increasing knowledge, attitudes, and hemoglobin levels due to anemia [31].

The study revealed that there is a significant enhancement by statistics (*p* < 0.01) after conducting interventions among adolescents in an intervention group and booklets. Another study revealed that the value of student efficacy and performance in behavior prevention increased by 3 months after intervention [32]. This result, supported by the results of a study from Cao et al., showed that people who use the application m-Health feel more satisfied [33,34,35,36]. The attitude of adolescents who are given intervention in the form of booklet after three months has the same effect as the WANTER method. This study is supported by Riyanti et al. that the use of the booklet has effectiveness in improving adolescent attitudes regarding the prevention of anemia [37,38].

The study shows there is no enhancement practice during three months of intervention. The reasons for the changing of behavior against anemia are multifactorial and complex. There are many possible factors that influence success in changing behavior, such as self-motivation, understanding in applying habits, and time of intervention. Moreover, an ideal time to improve behavior is six months or more [39]. The study conducted by Singh et al. explained that the most important strategy in prevention of anemia is knowledge and practices. Supported by research conducted by Ghosh at al., although there is good knowledge and a positive attitude, very few practices are applied [40]. This is different from the result of Susmita et al. that participants have a significant correlation among knowledge, attitude, and practice [41]. Several studies have stated that the adequate time to increase knowledge, attitudes, and practices is three months [42]. However, most studies explained that the adequate time is at least six months [39]. Therefore, we include this as a limitation. Accessibility to education in the form of information and social media could be obtained when just and where appropriate. Information that could influence the view and lifestyle depends on reliable sources [23]. The inability to access credible, reliable, and accurate information could impact negatively on knowledge by the population in general [24].

## 5. Limitation

The limitation of this study is the absence of an analysis of the increase in hemoglobin as an assessment on the incidence of anemia. In addition, we only intervened for three months, because the ideal time to improve behavior is six months or more.

## 6. Conclusions

Our study showed that adolescents’ knowledge and attitude increased significantly in three months after the intervention of WANTER and booklets toward preventing anemia. Knowledge, attitudes, and practices to prevent anemia need to be continuously improved. Health education through appropriate media for adolescents, by health workers, teachers, parents, and stakeholders, is very important to make interventions more effective.

## Figures and Tables

**Table 1:** Sociodemographic Characteristic.

Variables	Intervention		Control		Total		*p*-Value
	Median	Min-Max	Median	Min-Max			
**Age (in years)**	17	15–19	17	15–19			0.633
	**n = 162**	**%**	**n = 115**	**%**	**n = 277**	**%**	
**Educational stage**							
10th grades	79	48.77	65	56.52	144	51.99	0.223
11th grades	83	51.23	50	43.48	133	48.01	
**Living with/in**							
Dormitory	5	3.09	26	22.61	31	11.19	0.000
Family	157	96.91	89	77.39	246	88.81	
**Father’s education**							
Primary school	49	30.25	35	30.43	84	30.32	0.520
Secondary school	90	55.56	70	60.87	160	57.76	
University	23	14.20	10	8.70	33	11.91	
**Mother’s education**							
Primary school	50	30.86	41	35.65	91	32.85	0.256
Secondary school	91	56.17	64	55.65	155	55.96	
University	21	12.96	10	8.70	31	11.19	
**Father’s occupation**							
Employed	154	95.06	110	95.65	264	95.31	0.779
Unemployed	3	1.85	5	4.35	8	2.89	
Other (Died)	5	3.09	0	0.00	5	1.81	
**Mother’s occupation**							
Employed	37	22.84	24	20.87	61	22.02	0.604
Unemployed	125	77.16	90	78.26	215	77.62	
Other (Died)	0	0.00	1	0.87	1	0.36	
**Household monthly income**							
Less than 1 million	63	38.89	54	46.96	117	42.24	0.239
1–3 million	66	40.74	40	34.78	106	38.27	
More than 3 million	33	20.37	21	18.26	54	19.49	
**History of hemoglobin test**							
Ever checked	18	11.11	14	12.17	32	11.55	0.850
Never checked	144	88.89	101	87.83	245	88.45	
**History of taking blood tablets**							
Ever consume	139	85.80	88	76.52	227	81.95	0.570
Never consume	23	14.20	27	23.48	50	18.05	
**Anemia status**							
Anemia	24	14.81	14	12.17	38	13.72	0.597
Not anemic	138	85.19	101	87.83	239	86.28	
**Resources about IDA**							
Books	0	0.00	1	0.87	1	0.36	0.003
Internet	0	0.00	1	0.87	1	0.36	
Social media	73	45.06	24	20.87	97	35.02	
Public health center	6	3.70	13	11.30	19	6.86	
School	37	22.84	40	34.78	77	27.80	
Don’t know	46	28.40	36	31.30	82	29.60	
**Experience coming to health services**							
Yes	74	45.68	57	49.57	131	47.29	0.543
No	88	54.32	58	50.43	146	52.71	
**Purpose of coming to health services**							
Get treatment	27	16.67	15	13.04	42	15.16	0.785
Consultation	0	0.00	1	0.87	1	0.36	
Medical check-up	40	24.69	31	26.96	71	25.63	
Don’t know	95	58.64	68	59.13	163	58.84	

**Table 2:** Comparison of KAP between Control and Intervention Group.

Variable	Group	Before Intervention	3 Months after Intervention	*p* Value
		Median (Min–Max)	Median (Min–Max)	
Knowledge	Intervention (n = 162)	6 (0–8)	8 (0–9)	0.000
	Controls (n = 115)	6 (1–8)	8 (1–9)	0.000
	***p* value**	0.82	0.61	
Attitude	Intervention (n = 162)	0 (0–1)	2 (0–4)	0.000
	Controls (n = 115)	0 (0–1)	2 (0–4)	0.000
	***p* value**	0.9	0.22	
Practice	Intervention (n = 162)	3 (0–4)	3 (0–5)	0.091
	Controls (n = 115)	3 (1–5)	3 (0–5)	0.453
	***p* value**	0.12	0.1	

## Data Availability

Not applicable.

## Appendix A

Questionnaire of knowledge, attitude, and practice (KAP) from the Food and Agriculture Organization of the United Nations (FAO) [19].
